# A Review of the Main Biologically Active Compounds of the Genus *Echium* L., Naturally Distributed in Bulgaria, and Their Pharmacological Potential

**DOI:** 10.3390/ph18111618

**Published:** 2025-10-27

**Authors:** Svetoslava Terzieva, Neli Grozeva, Milena Tzanova

**Affiliations:** Department of Biological Sciences, Faculty of Agriculture, Trakia University, Students’ Campus, 6000 Stara Zagora, Bulgaria; n.grozeva@trakia-uni.bg (N.G.); milena.tsanova@trakia-uni.bg (M.T.)

**Keywords:** *Echium italicum* L., *Echium russicum* J.F. Gmel., Bulgaria, *Echium vulgare* L., *E. plantagineum* L., biological activity, pharmacology

## Abstract

Worldwide, the genus *Echium* L. (Boraginaceae) is represented by over 60 species of herbaceous plants and shrubs. The species are widely distributed all around the Mediterranean basin, Europe, and the Macaronesian Islands and are known for their analgesic, diuretic, antioxidant, antimicrobial, and antitumor properties. In traditional medicine, they are widely used as a wound-healing and anti-inflammatory agent, for respiratory problems and problems related to mental health, and for general abrasions and fissures of the hands. Four species are naturally distributed in Bulgaria—*E. russicum* J.F. Gmel., *E. vulgare* L., *E. italicum* L., *E. plantagineum* L., the first three being medicinal. The review aims to summarize the literature describing the content of biologically active substances and the therapeutic effects of *Echium* spp., with an emphasis on medicinal species distributed in Bulgaria. The content of biologically active substances was monitored, in particular, terpenes, phenolic compounds, flavonoids, naphthoquinones, omega-3 and omega-6 fatty acids, and pyrrolizidine alkaloids. The relationship between bioactive compounds, biological activities, and medicinal uses was researched. After the analysis made in the present review, it can be summarized: Despite extensive research, knowledge of their pharmacological potential is still incomplete. An attempt has therefore been made to outline directions for future research.

## 1. Introduction

The genus *Echium* L. (Boraginaceae) comprises more than 60 annual, biennial, or perennial herbaceous species and subshrubs, predominantly distributed across the Mediterranean region, Europe, and the Macaronesian Islands [1,2]. Some species have also been introduced and naturalized in South Africa, North America, and Australia since the late 19th century [3,4].

Species from the genus *Echium* have long been used both ornamentally and medicinally. The increasing scientific interest in *Echium* spp. stems from their rich and diverse phytochemical profile, including pyrrolizidine alkaloids (PAs), phenolic compounds, flavonoids, and polyunsaturated fatty acids such as γ-linolenic acid (GLA) [5,6]. These bioactive compounds exhibit confirmed antioxidant, anxiolytic, anti-inflammatory, antimicrobial, and cytotoxic properties (anticancer and antitumor compounds), making these plants promising candidates for pharmaceutical development [7,8,9,10,11].

In traditional medicine, extracts from *Echium* spp. are employed in conditions associated with oxidative stress and neuropsychiatric disorders—such as anxiety, depression, epilepsy, ischemic stroke, and obsessive-compulsive disorder—as well as for the treatment of surface wounds, skin fissures, and insect and snake bites [12,13,14]. The therapeutic efficacy of these plants is largely attributed to polyphenolic compounds and unsaturated fatty acids, mainly extracted from seeds but also found in the leaves [15,16,17]

However, the presence of PAs necessitates a careful toxicological evaluation due to their known hepatotoxic and potentially carcinogenic effects [18,19].

This underscores the importance of assessing both the therapeutic potential and safety of the species of genus *Echium* before considering them for broader pharmaceutical or nutraceutical applications.

Beyond their medicinal relevance, *Echium* spp. also serve ecological functions as valuable nectar sources for pollinators, particularly honeybees (*Apis mellifera*), which further increases interest in their cultivation and conservation [20,21,22]. The exploration of bioactive compounds within this genus could facilitate the development of new natural products, including herbal supplements, phytopharmaceuticals, and cosmetic formulations, aligning with the global demand for plant-based and sustainable alternatives to synthetic drugs.

In Bulgaria, four *Echium* species are recorded—*E. italicum* L., *E. russicum* J.F. Gmel., *E. vulgare* L., and *E. plantagineum* L.—the first three of which are included in the Medicinal Plants Act in Bulgaria (State Gazette No. 29/2000) [23]. Despite the growing interest in the potential pharmaceutical uses of *Echium* spp., these species remain relatively underexplored in terms of their chemical composition and pharmacological value.

This study aims to systematically review and analyze the major bioactive compounds found in *Echium* species naturally distributed in Bulgaria, with particular attention to interspecific differences in phytochemistry and biological activity. It further evaluates their potential applications and offers guidance for future phytochemical and pharmacological research, highlighting species with the greatest promise for pharmaceutical development and sustainable use.

## 2. Materials and Methods

This study was conducted as a systematic literature review focused on the phytochemical composition and biological activity of species belonging to the genus *Echium*, with an emphasis on the four species recorded in the territory of Bulgaria: *E. italicum*, *E. russicum*, *E. plantagineum*, and *E. vulgare*.

**Data Sources and Search Strategy**

A structured literature search was performed using the following international scientific databases: Google Scholar, ScienceDirect, PubMed, Web of Science, and CABI. The search included peer-reviewed publications from the period 2000 to 2025. The following search terms and keyword combinations were used: “*Echium* L.”, “*Echium italicum*”, “*Echium russicum*”, “*Echium plantagineum*”, “*Echium vulgare*”, “bioactive compounds”, “phytochemical composition”, “pharmacological activity”, “medicinal use”, “plant constituents”, “pharmaceutical applications”. Boolean operators (AND, OR) were used to optimize search combinations.

**Inclusion Criteria**

Sources were selected for inclusion based on the following criteria:

- The publication is a peer-reviewed scientific article, review, dissertation, or academic report;
- Written in English or Bulgarian;
- Contains relevant keywords in the title, abstract, keywords section, or full text;
- Provides information on phytochemical composition, biological activity, or medical/pharmaceutical application of any of the target *Echium* species.

**Data Extraction and Analysis**

The selection process included:

- Preliminary screening of titles and abstracts;
- Full-text review of eligible articles. Extraction and tabulation of relevant data, including: identified bioactive compounds, reported pharmacological activities, and toxicological profiles, where available;
- Comparative analysis of the phytochemical profiles and documented applications of the Bulgarian *Echium* species.

Studies were excluded if they: Focused exclusively on *Echium* species not naturally distributed in Bulgaria, without providing comparative data relevant to the target taxa.

All data were categorized by species and analyzed to identify commonalities and differences in their chemical constituents and therapeutic potentials. This approach aimed to provide a synthesized overview of the current knowledge and to identify promising candidates for further investigation in pharmacology and applied phytotherapy.

The structural formulas of the chemical compounds included in this review were prepared using ACD/ChemSketch, version (2015), Advanced Chemistry Development, Inc., Toronto, ON, Canada.

## 3. Botanical Description of *Echium* Spp. Distributed in Bulgaria

The genus *Echium* L. (Boraginaceae) is represented in the Bulgarian flora by four species: *E. italicum*, *E. russicum*, *E. plantagineum*, and *E. vulgare*. These species are annual, biennial, or short-lived perennial herbaceous plants characterized by erect, hispid stems bearing appressed and spreading bristles (Table 1). Plants typically reach 30–150 cm in height, with inflorescences composed of coiled cymes and five-lobed corollas that change color during anthesis, usually from reddish to blue, violet, or whitish hues. The fruit is a schizocarp, generally splitting into four ovoid-triquetrous nutlets (mericarps), enclosed by persistent bristly sepals [24,25]. The species are mostly associated with open, grassy, stony, or ruderal habitats across the country, from lowlands up to 1500 m a.s.l.

### 3.1. Echium italicum

A biennial species with stems 40–150 cm tall, densely covered with appressed hairs and bristly tuberculate trichomes. Basal leaves are lanceolate, 20–35 × 1.5–4.0 cm, with appressed bristles; cauline leaves are narrower and elliptic. Inflorescences form a symmetrically branched pyramidal panicle with yellowish, pinkish, or bluish-white flowers. The calyx measures 6–7 mm; corolla 10–12 mm, narrowly funnel-shaped, whitish to blue-violet, with 4–5 long-exserted stamens. Nutlets are ovoid-triquetrous, 2.8–3.8 × 2.3–2.5 mm. Flowering May–June, fruiting July–September. Found in grassy and stony sites, roadsides, and ruderal habitats up to 1000 m a.s.l. [26,27,28].

Conservation status: Not protected under the Bulgarian Biodiversity Act (2002) [29].

### 3.2. Echium russicum

A biennial herb with solitary stems (25–80 cm tall), densely leafy and hispid. Leaves are linear-lanceolate, 2–11 × 0.7–1.2 cm. Inflorescences are spike-like, occupying up to half the stem, and composed of coiled cymes. Corolla 8–12 mm, funnel-shaped, dark red; stamens and style exserted; stigma entire or shallowly bifid. Nutlets 1.8–3.2 × 1.5–1.8 mm. Flowering May–June; fruiting June–July. Inhabits dry meadows and rocky shrublands up to 1200 m a.s.l. [27,28].

Conservation status: Listed as Vulnerable in the Red Data Book of Bulgaria [30] and protected under the Bulgarian Biodiversity Act (2002) [29].

### 3.3. Echium plantagineum

An annual to biennial herb with branched stems 20–60 cm tall, covered with bristles. Basal leaves ovate, 3–12 × 1.2–1.5 cm; cauline leaves narrow-lanceolate, cordate at base, bristly with bulbous bases. Inflorescences are broad, paniculate, or racemose. Corolla 15–20 mm, blue to deep blue (rarely white), sparsely hairy externally. Two stamens are shortly exserted; three are included. Nutlets 2.0–3.0 × 2.0–2.3 mm. Flowering May–June; fruiting July–August. Typically found in grassy, stony, and ruderal habitats, usually below 1000 m a.s.l. [27,28,31].

Conservation status: Not protected under the Bulgarian Biodiversity Act (2002) [29].

### 3.4. Echium vulgare

A biennial to short-lived perennial herb with stems 20–100 cm tall, branched, hispid with two types of hairs: stout spreading bristles with pustulate bases, and fine appressed hairs. Older hairs become brittle and prickly, arising from red to black tubercles speckling the stem. Stem leaves alternate, sessile, linear-lanceolate; rosette leaves 2–25 × 0.5–3 cm, oblanceolate, entire, shortly petiolate. Calyx deeply 5-lobed, hispid; corolla pink in bud, bright blue at anthesis (sometimes white or pink), turning purplish to brownish with age, 10–20 mm, zygomorphic. Inflorescences are panicles of helicoid cymes, each bearing ~20 flowers; up to 50 cymes per stem. Style bifid, branches >1 mm. Nutlets 1.8–3.2 × 1.5–1.8 mm, pointed at tip, with flattened basal scar. Seedlings develop contractile taproots; cotyledons ~1 cm, ovate, pubescent [32]. Flowering May–July; fruiting July–October. Common in grasslands, rocky slopes, ruderal sites, and along roadsides up to 1500 m a.s.l. [27,28].

Conservation status: Not protected under the Bulgarian Biodiversity Act (2002) [29].

## 4. Ethnopharmacological Use of *Echium* Species in Folk Traditional Medicine

The use of *Echium* spp. outside conventional medicine has been documented in several ethnobotanical studies covering regions of Europe, the Caucasus, the Middle East, and North Africa (Table 2). These species are traditionally known for their anti-inflammatory, expectorant, emollient, and wound-healing properties, making them valuable in the treatment of a broad spectrum of health conditions [33]. Iranian *Echium* species, collectively known as *Gol-e-Gavzaban*, are native plants widely used as food and in traditional remedies [34].

### 4.1. Echium italicum

Distributed predominantly in Southern Europe, *E. italicum* has found use in Italian and Greek folk medicine, especially in the treatment of respiratory ailments (Table 2). A decoction of the aerial parts is consumed as a tea for sore throat and cough, and in some regions it is also applied as a mild diuretic [35]. In Turkey, known as *Kanar otu*, it is used topically for abscesses, boils, wounds, and snake bites, often as an oil macerate from the roots [36,37,38,40,41,42]. In Italy, infusions are used for their depurative, diaphoretic, diuretic, and emollient properties [39]. A paste made from roasted root and oil is traditionally applied after injury to support wound-healing. Ethnobotanical data suggest a primary role in managing respiratory tract infections.

### 4.2. Echium russicum

Although relatively underexplored, *E. russicum* has been recorded in the traditional medicinal practices of Turkey, where its roots are employed for external applications [36]. In folk medicine, the species has been used for wound-healing, osteomyelitis, and hemorrhoids (Table 2). Moreover, it has been reported as a remedy against snake bites, while the leaves have been employed in gynecology, particularly in the postnatal period [44]. Additionally, in certain locales, wine prepared from its flowers is traditionally considered a restorative agent for anemia and fatigue [43].

### 4.3. Echium plantagineum

This species is widely used across the Mediterranean (Table 2). In Portugal and Spain, it is known as *viperina*, and is traditionally applied as an expectorant and diuretic, also for urinary tract infections, fever, inflammation, and muscle strain [13]. In Morocco and Tunisia, dried flowers are mixed with honey to treat sore throats and coughs [45]. The aerial parts are often prepared as a diaphoretic herbal tea [33].

### 4.4. Echium vulgare

One of the most thoroughly documented species, *E. vulgare* (commonly known as “Viper’s Bugloss” or “Blueweed” in English and “Havaciva” in Turkish), has been traditionally used for wound-healing, bruises, muscle and ligament strains, and sprains. Its root was often prepared as an ointment by boiling it in oil [13,36,41,46]. Leaves and flowers have been applied as diuretics and antitussives. The infusion (tea) of *E. vulgare* exhibits expectorant and laxative properties and has traditionally been used in the treatment of diarrhea in Serbia [47]. Ethnobotanical reports also mention its use against snake bites and scorpion stings [48,49]. In the Balkan Peninsula (Bulgaria, North Macedonia, Croatia, and Montenegro), both *E. vulgare* and *E. italicum* are used for making infusions to treat colds, and externally for wounds, boils, insect bites, and burns. Local names such as “zmiyska treva” (snake herb), “zhaburnik”, and “koshnichka” reflect folk beliefs in their antidotal and soothing powers [24,42]. In Sefrou Province, Morocco, different parts of *E. vulgare* are traditionally consumed orally, with leaf infusions used as depuratives, flower infusions as balsamic agents, and seed infusions as lactogenic remedies [50]. Additionally, *E. vulgare* has been used for gynecological disorders [51].

## 5. Phytochemical Profile of Studied *Echium* Species

**Figure 1:**
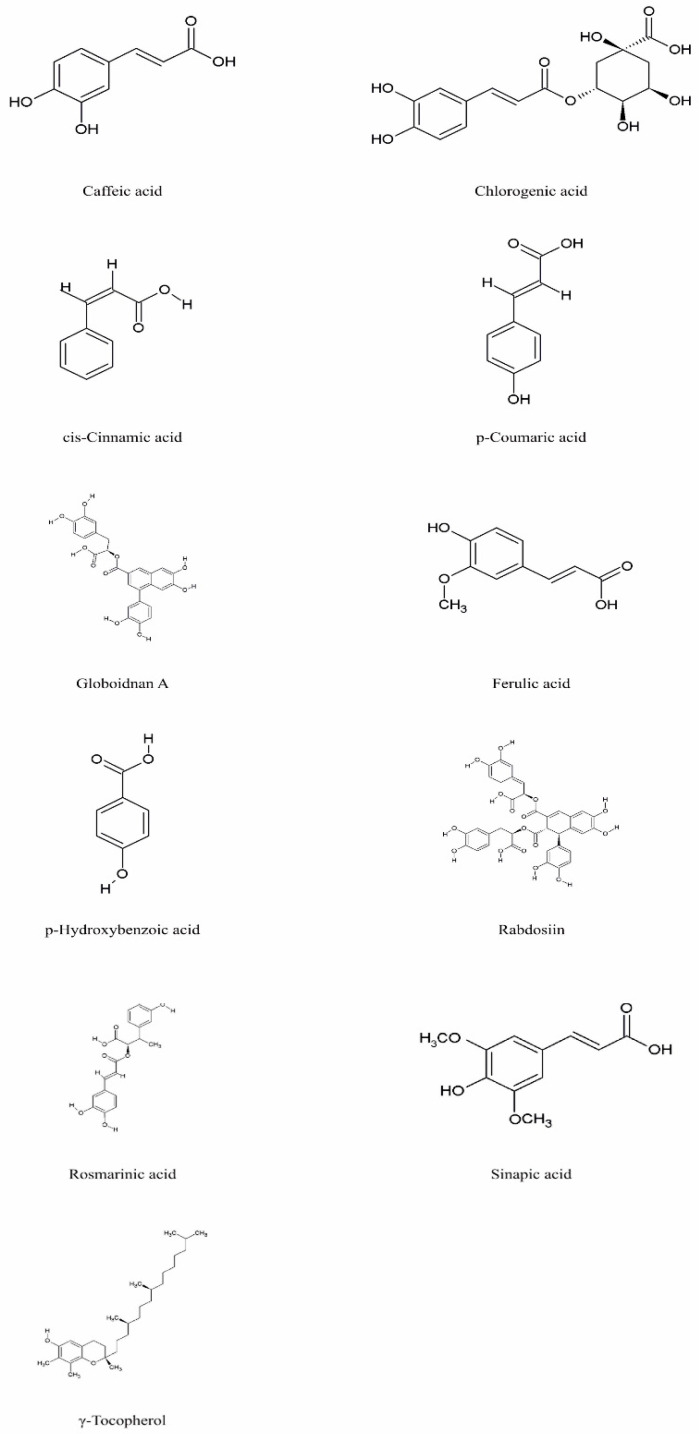
Main phenolic compounds in *Echium* spp.

**Figure 2:**
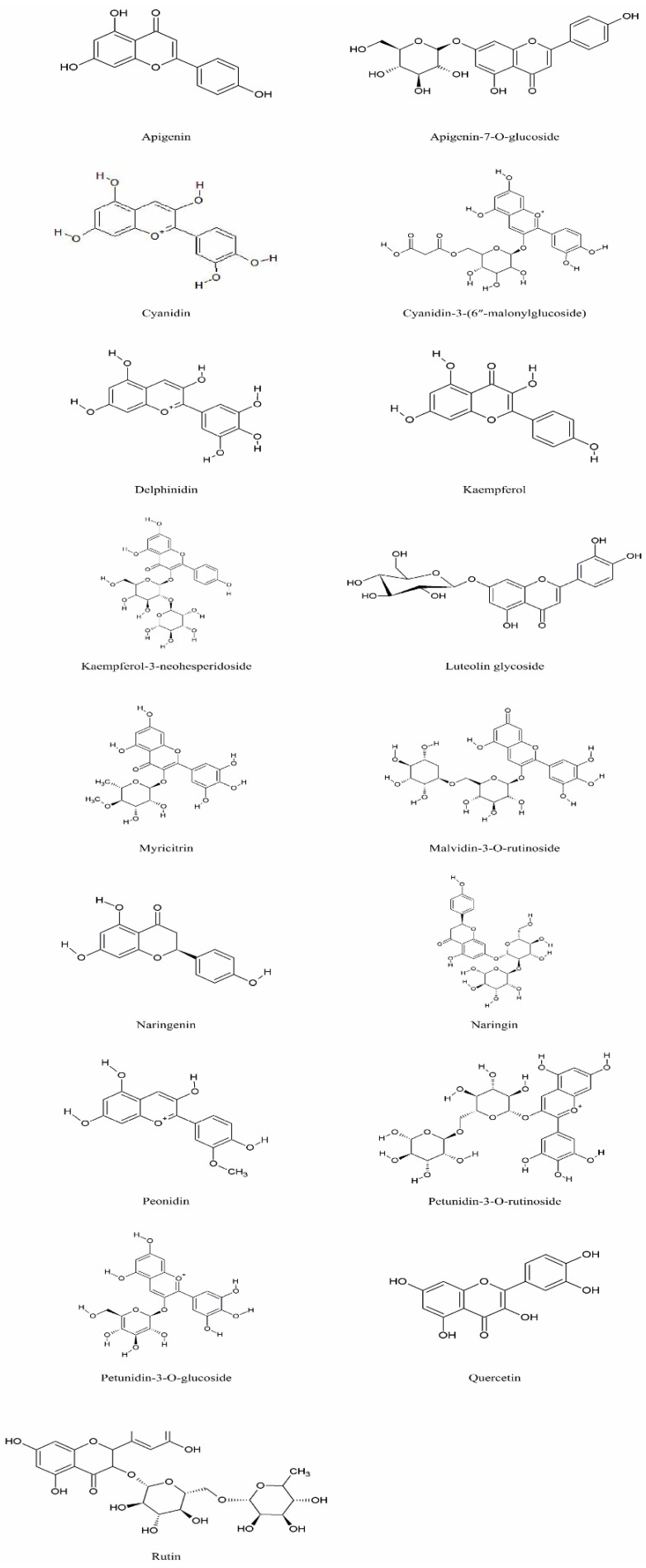
Main flavonoids in *Echium* spp.

**Figure 3:**
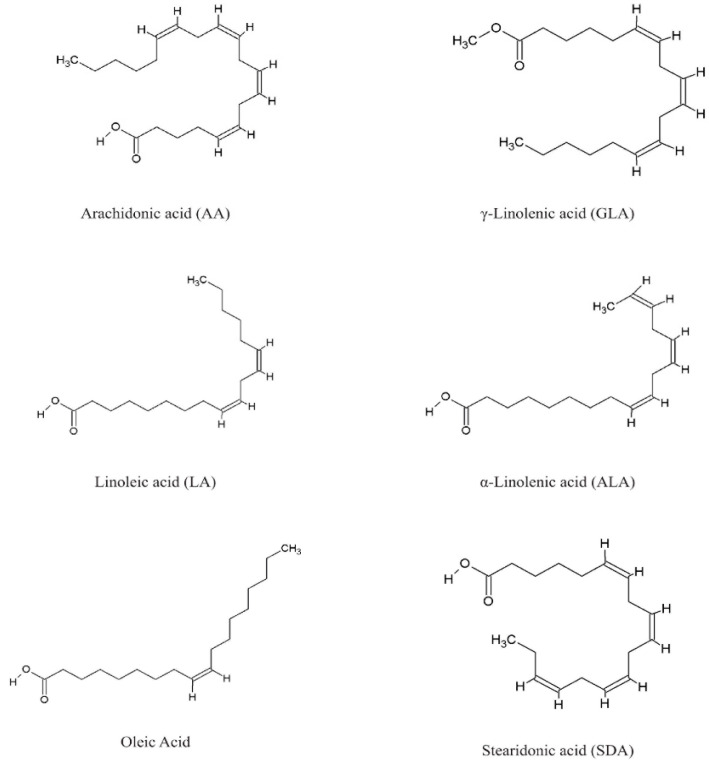
Main fatty acids in *Echium* spp.

**Figure 4:**
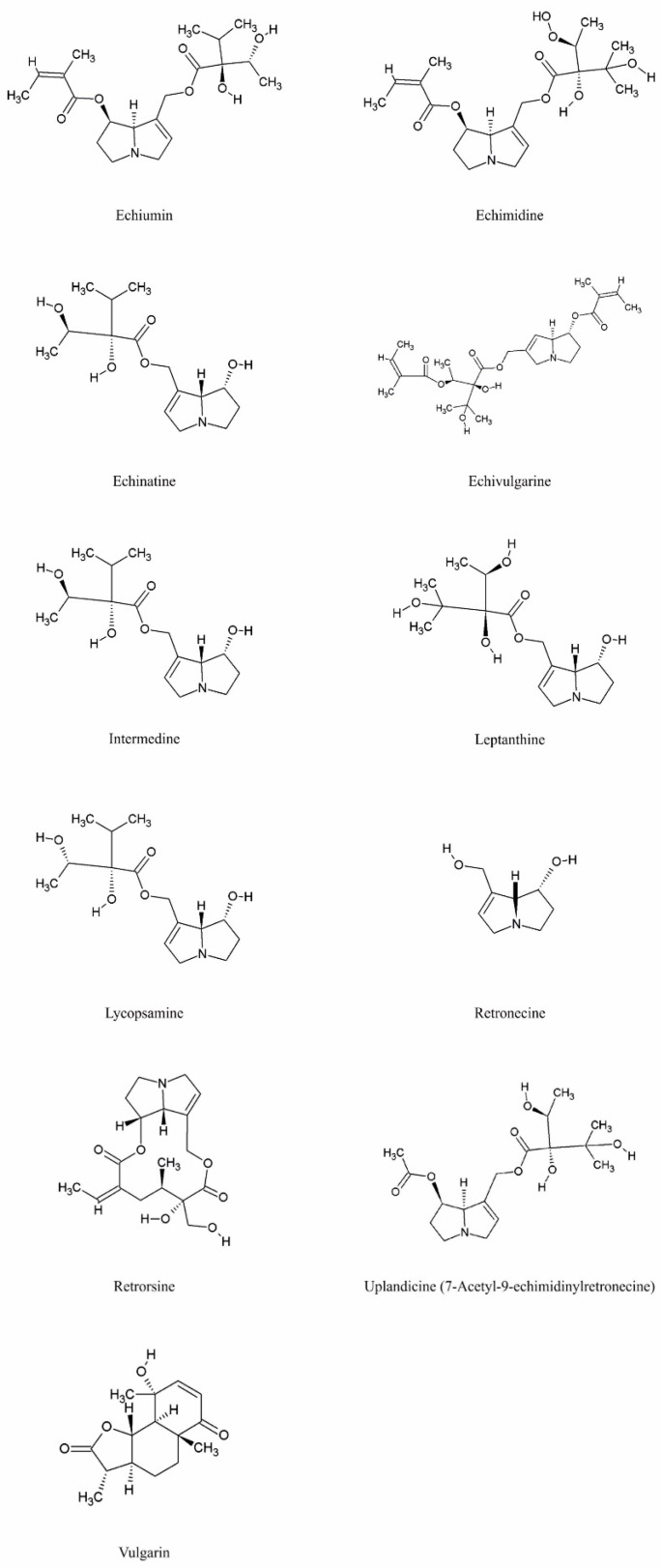
Main pyrrolizidine alkaloids in *Echium* spp.

**Figure 5:**
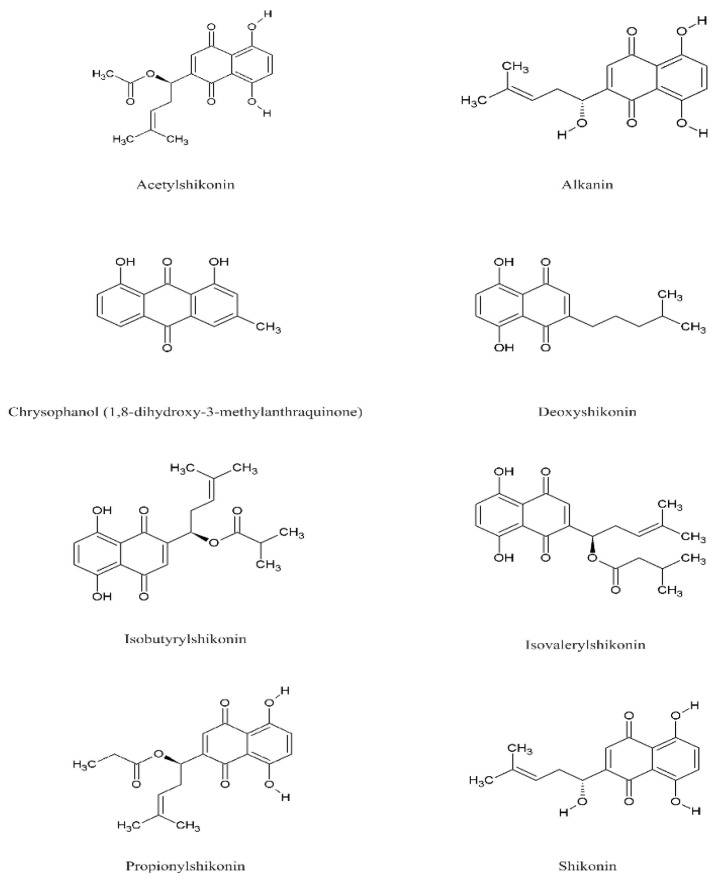
Main quinones in *Echium* spp.

**Figure 6:**
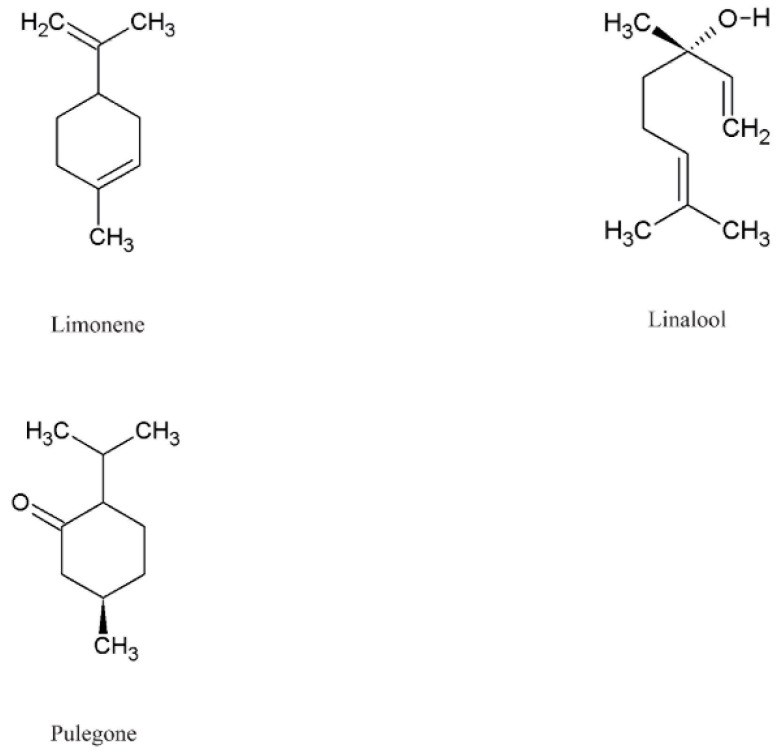
Main terpenes in *Echium* spp.

**Figure 7:**
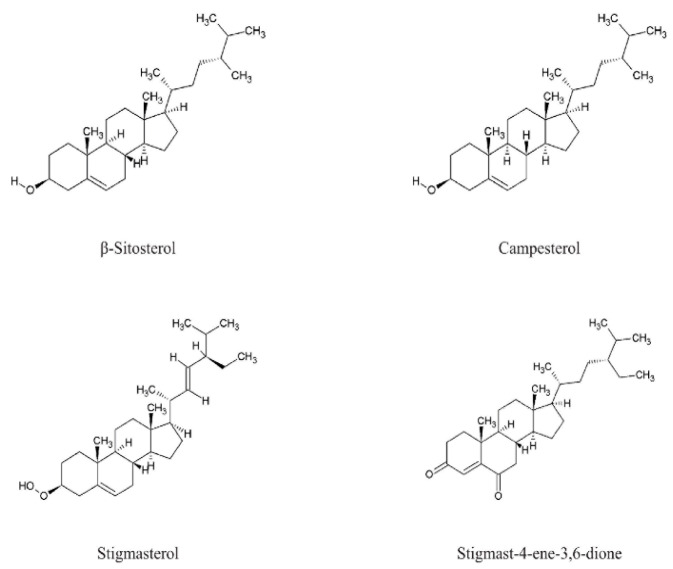
Main phytosterols in *Echium* spp.

Species of the genus *Echium* contain a diverse secondary metabolites with significant physiological and pharmacological relevance. Among the most studied bioactive compounds isolated from representatives of the genus are phenolic compounds, flavonoids, polyphenolic acids, pyrrolizidine alkaloids, sterols, essential fatty acids—including γ-linolenic acid (GLA)—as well as essential oils and saponins [52,53]. Biological activities of phytochemicals isolated from *E*. *italicum*, *E. russicum*, *E. plantagineum*, and *E. vulgare* are illustrated in Table 3, and individually isolated phytochemicals from these plants are listed in Figure 1, Figure 2, Figure 3, Figure 4, Figure 5, Figure 6 and Figure 7.

*Echium* species remain insufficiently characterized in terms of their phytochemical profiles, and in vivo mechanistic studies are still lacking [33].

### 5.1. Phenolic Compounds

Phenolic acids are among the most important phytochemicals in *Echium* species, playing a key role in their antioxidant, UV-protective, and pharmacological properties. Across different *Echium* species, the main phenolic acids identified include rosmarinic acid, cis-cinnamic acid, caffeic acid, chlorogenic acid, caffeoylquinic acid, p-hydroxybenzoic acid, p-coumaric acid, and ferulic acid [54,58,74,96,116]. The chemical structures of the main phenolic acids identified in *Echium* spp. are shown in Figure 1, while their reported biological activities are summarized in the text and Table 3.

Rosmarinic acid is the most widespread phenolic acid, detected in all investigated species, and is well known for its antioxidant and anti-inflammatory activities [40,116].

Acetone extracts of *E. italicum* were reported to contain various polyphenols such as p-hydroxybenzoic acid, chlorogenic acid, p-coumaric acid, ferulic acid, and sinapic acid, as well as flavonoids (which belong to the large polyphenolic family), suggesting a potential contribution to the antimicrobial properties of the species [55]. Similarly, in *E. altissimum* Jacq. (syn. *E. italicum*), methanolic extracts revealed rosmarinic acid and myricitrin as major constituents, highlighting their potential as natural antimicrobial agents in pharmaceutical applications [56].

Investigations of the underground organs of *E. vulgare* and *E. russicum* demonstrated the presence of rosmarinic acid in their roots [71,72]. In *E. vulgare* seeds, 15 phenolic acids were identified, with caffeic, salicylic, p-hydroxybenzoic, and 3,4-dihydroxybenzoic acids being the most abundant [97]. Furthermore, methanolic extracts of *E. vulgare* were found to contain high levels of gallic acid, benzoic acid, and isoferulic acid, indicating a strong potential for antioxidant and pharmacological applications [98].

Boskovic et al. [99] demonstrated high concentrations of pharmacologically active tannins in chloroform extracts, a trend similarly observed in *E. italicum* through chloroform and acetone extracts [37]. According to Taravati et al. [54], the level of tannins in the tissues of different *Echium* spp. varies depending on environmental stresses.

### 5.2. Flavonoids

Flavonoids, along with phenolic acids, are among the most abundant phytochemicals in the aerial parts of *Echium* species and play a significant role in their antioxidant activity by scavenging free radicals [6,117]. The main flavonoids identified across various species include quercetin, luteolin, kaempferol, rutin, luteolin glycoside, apigenin glycoside, myricitrin, naringenin, and anthocyanins such as petunidin, delphinidin, peonidin, cyanidin, and malvidin [55,58,67,100] (Figure 2).

In *E. italicum* and *E. vulgare*, flavonoid concentrations are generally higher in the aerial parts, whereas the roots tend to contain greater total phenolic content [45,101]. The choice of solvent significantly influences the composition and bioactivity of the extracts. For example, ethanol extracts of *E. vulgare* exhibited the highest phenolic and flavonoid contents, alongside stronger antimicrobial and antioxidant activity compared to chloroform extracts, and similar trends were observed in *E. italicum* [37,99].

Recent studies on aqueous and ethanolic extracts from the aerial parts of *E. vulgare* performed both qualitative and quantitative analyses of bioactive compounds. Using the aluminum chloride spectrophotometric method, the total flavonoid content was determined to be 2.59%, with a maximum extract yield of 16% [103]. These findings highlight the rich flavonoid composition of *Echium* species, emphasizing their potential contribution to antioxidant and antimicrobial properties.

Aqueous and methanolic extracts from the aerial parts of *E. plantagineum* collected in Algeria exhibited strong antioxidant activity, which was attributed to their high phenolic content [118]. Pollen analysis of the same species revealed eight anthocyanin pigments, with petunidin-3-O-rutinoside as the predominant pigment, followed by delphinidin, cyanidin, and malvidin derivatives, further supporting the phytochemical richness of this species [67].

### 5.3. Fatty Acids

Species within the genus *Echium* have garnered increasing scientific interest due to their seed oil composition, which is rich in nutritionally and pharmacologically valuable polyunsaturated fatty acids, particularly γ-linolenic acid (GLA) and stearidonic acid (Figure 3). Notable interspecific and geographic variability in fatty acid profiles suggests both considerable biochemical diversity and significant adaptive potential, underscoring the taxonomic and biotechnological relevance of this group [76].

The data of seeds of various *Echium* species, collected from their natural habitats in Turkey, were analyzed by gas chromatography to determine their total oil content and detailed fatty acid composition. The fatty acid profiles—including relative concentrations and the presence of unusual fatty acids—may serve as effective biochemical markers for taxonomic differentiation at both the genus and subgenus levels within Boraginaceae [119,120,121]. All studied taxa exhibited high proportions of linoleic acid and α-linolenic acid, two essential fatty acids known to support human health and meet reference dietary intake recommendations. According to Bilgiç-Keleş et al. [104], *E. vulgare* seed oil is particularly rich in stearidonic acid, showing higher levels compared to other species in the genus.

From a nutritional and dermatological perspective, *E. plantagineum* is distinguished by its high content of omega-3 and omega-6 fatty acids as GLA, stearidonic acid SDA, and α-linolenic acid (ALA), making it a valuable raw material for nutraceutical applications [53,122]. Several *Echium* species, including *E. vulgare* and *E. plantagineum*, were found to be particularly rich in unsaturated fatty acids such as GLA and ALA [76,77,78,79,80,105]. GLA, in particular, is recognized for its anti-inflammatory properties and is widely employed in dietary supplements and dermatological formulations. The seed oil of these species may contain approximately 12–14% GLA, making them competitive with commercial sources such as *Oenothera biennis* L. (evening primrose), *Borago officinalis* L. (borage), and *Ribes nigrum* Marshall (blackcurrant) [73,81,123,124]. Among the highest reported seed oil yields are those of *B. officinalis* and *E. vulgare*, with oil contents of 28.3% and 34.7%, respectively [106].

In addition to its overall lipid profile, *E. vulgare* seeds exhibit dynamic changes in fatty acid composition during maturation, characterized by increasing levels of α-linolenic (ALA) and stearidonic (SDA) acids and a parallel decline in γ-linolenic (GLA) and linoleic (LA) acids [107]. Slight quantitative differences were observed between plant organs. The aerial parts contained higher total levels of essential and semi-essential amino acids.

Fatty acid analysis across additional *Echium* species further confirmed the nutritional significance of this genus. GLA content of up to 10.9% was detected in *E. vulgare*, consistent with other continental species, while *E. russicum* exhibited up to 15.8% GLA. The elevated GLA levels observed in *E. russicum* are likely influenced by its habitat in the Macaronesian region, where high GLA content appears to be a common trait among endemic *Echium* taxa [15,82]. Several *Echium* species also accumulate high levels of SDA, positioning them as promising plant-based sources of omega-3 fatty acids [125].

*Echium plantagineum* seeds, in particular, possess a highly unsaturated oil profile, containing approximately 14% linoleic acid (LA), 10% GLA, 33% ALA, and 14% SDA [83]. Comparable results were reported by Kavanagh et al. [84], who found that *Echium* seed oil has a pleasant odor and taste and contains 12–14% of its fatty acids as SDA (n-3) and 9–11% as GLA (n-6). The high concentrations of ALA and SDA render this species a valuable candidate for dietary supplementation and therapeutic applications. Recent studies further support its potential in the management of inflammatory disorders, cardiovascular diseases, and cancer [75,80,85,126,127].

Analysis of oil from seeds of *Echium* spp., including *E. italicum* and *E. russicum*, determined different fatty acids, including linolenic acid, linoleic acid, oleic acid, and arachidonic acid as major fatty acids, while stearic acid, palmitic acid, and GLA were the minor fatty acids extracted from seeds [54]. Although seeds are the primary site for fatty acid accumulation, other plant organs, including leaves and stems, also contribute to overall fatty acid content. In *E. italicum*, total unsaturated fatty acid levels were highest in seeds, with polyunsaturated fatty acids predominating over monounsaturated and saturated fatty acids. The LA: ALA ratio varied considerably depending on species, plant organ, and geographic origin—highlighting ecological adaptability and offering potential for breeding programs [59].

In-depth chemical analyses of *E. italicum* revealed α-linolenic, linoleic, oleic, stearidonic, and γ-linolenic acids as the main unsaturated fatty acids. Notably high levels of SDA and GLA were recorded in seed oil from Turkish populations [60,61]. Reported ranges for oil components in *E. italicum* include total oil content (6.2–28.4%), GLA (0.61–2.19%), SDA (3.94–9.79%), palmitic acid (6.51–18.93%), stearic acid (3.67–4.30%), oleic acid (12.63–16.23%), LA (14.09–20.15%), and ALA (22.12–36.61%) [61,62].

### 5.4. Pyrrolizidine Alkaloids

Pyrrolizidine alkaloids (PAs) are common secondary metabolites in the Boraginaceae family and are well known for their toxicity, particularly hepatotoxic effects [52,128] (Figure 4). Despite their toxic potential, these compounds also exhibit antimicrobial and cytotoxic activities and play a crucial role in *Echium* species as a defense mechanism against herbivores and pathogens. They have been extensively studied, including analyses of pollen composition [109,129,130,131].

These alkaloids are primarily synthesized in the roots and subsequently transported to the aerial parts of the plant. Identified in *Echium* species include echiiine, echimidine, intermedine, and their acetylated derivatives [86]. Stefova et al. [63] reported that among the prevalent Boraginaceae species in Macedonia, *E. vulgare* exhibits a higher toxic potential (up to 1340 mg/kg) compared to *E. italicum* (up to 16 mg/kg). Concentrations vary depending on species, developmental stage, and environmental conditions, with the highest levels generally found in the aerial biomass and seeds. In *E. vulgare* and *E. plantagineum*, PAs content is higher than in other studied species, and in *E. plantagineum*, levels can reach up to 0.4% of dry weight in aerial parts, correlating with the species’ ability to respond to abiotic stress [87,88,132,133].

In the anthers of *E. vulgare*, PAs are mainly present as N-oxides. The principal alkaloid in pollen is echivulgarine, along with vulgarine, 7-O-acetylvulgarine, and their respective N-oxides [110,111,112]. Similar findings were reported for the entire aerial part of *E. vulgare* by Mädge et al. [113]. These data highlight the diverse distribution and toxicological significance of PAs in *Echium* species, emphasizing their dual role in plant defense and potential health risks.

### 5.5. Quinones

Characteristics of the genus *Echium* are naphthoquinone pigments—shikonin and alkanin—and their esters (Figure 5). These compounds have demonstrated wound-healing, antimicrobial, and antitumor activities [37,134]. They are characterized by potent antifungal, anticancer, antidiabetic, and neuroprotective activities [135]. In addition to their beneficial properties, naphthoquinones and anthraquinones exhibit toxicological effects, mainly due to their presence as photoproducts in air pollutants [136].

Shikonin and its derivatives, isolated from *Echium* spp., have numerous applications in food, cosmetics, and textiles. Shikonin, a powerful bioactive red pigment, is well known for its diverse pharmacological potential [137,138]. Besides therapeutic effects, the naphthoquinone pigments shikonin and alkanin, found in *E. vulgare*, can cause gastroenteritis and diarrhea [32].

The underground organs of *Echium* spp. are rich in shikonins and their derivatives. In studying *E. vulgare* and *E. plantagineum*, results are similar, with both species producing high levels of shikonins and their derivatives (iso-valeryl shikonin, dimethylacryl shikonin, shikonin, acetyl shikonin), with concentrations approximately 2.5 times higher in *E. vulgare* [90,91].

Young underground organs of *Echium* spp. produce larger amounts of anthra- or naphthoquinones, among which shikonin, alkanin, and acetylshikonin are frequently detected [37]. Shikonin is one of the phenolic compounds produced from *E. italicum* [64]. In the root epidermis of *E. italicum* L., nine shikonin pigments were identified: shikonin, acetyl shikonin, propionyl shikonin, iso-butyryl shikonin, 3,3-dimethylacryl shikonin, angeloyl shikonin, 2-methyl-n-butyryl shikonin, deoxy shikonin, and iso-valeryl shikonin [41,57,65,66,92]. This makes *E. italicum* a potential new source of shikonin and its derivatives for industrial use [68].

High levels of quinones have been detected in *E. plantagineum* [15,93,132]. Ethanol extracts from young root periderm tissues of *E. plantagineum* are bright red or pink and contain several unusual naphthoquinones, including acetyl shikonin and 1,3-dihydroxy-3-methyl anthraquinone, whereas mature tissues are colorless and contain 1,3-dihydroxy-3-methyl anthraquinone [94]. Shikonin derivatives, particularly acetyl shikonin, have also been found in the rhizodermis and leaves of the species [89]. Therefore, *E. plantagineum* could be used to enhance the production of potential pharmaceutical compounds such as acetylshikonin [95].

### 5.6. Terpenes

The essential oil content in *Echium* species is generally low compared to other genera, with mono- and sesquiterpenes such as 1,8-cineole, camphene, and linalool commonly detected [139] (Figure 6). These compounds have been reported to exhibit moderate antimicrobial and anti-inflammatory properties, contributing to the medicinal potential of the genus. For instance, hydrodistilled essential oil from *E. italicum* was analyzed by GC and GC-MS, revealing pulegone in a content of 8.8% as a major terpene [69]. A study conducted on *E. vulgare* reported that the essential oil of this species contains linalool, camphor, endo-borneol, α-terpineol, trans-geraniol, lavandulyl acetate, cis-geranyl acetate, trans-geraniol acetate, caryophyllene oxide isomers, and α-bisabolol, with linalool, α-terpineol, and trans-geraniol identified as the predominant constituents [114]. Hence, *E. vulgare* may represent a valuable natural source of these bioactive compounds. Furthermore, the essential oil of *E. vulgare* holds promise as a potential ingredient in cosmetic and perfumery formulations.

Overall, while the essential oil content is relatively low, the diversity of terpenoid compounds in *Echium* species supports their potential in pharmacological and agricultural applications. Further studies focusing on Bulgarian populations are warranted to better characterize these bioactive constituents and their ecological and medicinal roles.

### 5.7. Phytosterols

Phytosterols such as β-sitosterol, stigmasterol, and campesterol have been identified in extracts of *E. italicum* and *E. vulgare* [6,34,70] (Figure 7). These sterols are known for their vasodilatory, hypocholesterolemic, and antitumor activities. Notably, Pardo et al. [115] reported the presence of stigmast-4-ene-3,6-dione, a rare phytotoxic sterone isolated from the roots of *E. vulgare*, which acts as a steroidal plant growth regulator with notable growth-promoting and anti-stress effects.

## 6. Pharmacological Activity and Medical Applications

The phytochemical diversity of *Echium* species underlies their broad pharmacological activity, including antioxidant, anti-inflammatory, anxiolytic, antimicrobial, and cytotoxic effects. *Echium* spp., including *E. italicum* and *E. vulgare*, demonstrate remarkable wound-healing activity [41,46]. Extracts from *E. vulgare* contain high levels of phenolic compounds and flavonoids that improve blood parameters, lipid profiles, liver functions, as well as histopathological changes in the heart and liver [98]. Stearidonic acid and γ-linolenic acid, as unusual fatty acids, are of great importance from nutraceutical and biomedical perspectives. Specifically, SDA from the omega-3 series is a terrestrial alternative to marine sources. The creation of a comprehensive database involving extensive screening of *E. italicum* L. populations across various habitats in Anatolia will serve as a reference gene bank for the development of omega-3-rich cultivars and nutraceutical products for human nutrition and health [61]. Shikonin, derived from *E. italicum,* exhibits anticancer, antibacterial, and wound-healing properties [57]. It also demonstrates antihistaminic activity and is suitable for the treatment of major allergic diseases. Furthermore, it can be used as a colorant in the pharmaceutical, cosmetic, and textile industries [64].

A summary of the pharmacological activities reported for various *Echium* species, including extract types, experimental models, and observed effects, is presented in Table 4.

### 6.1. Antioxidant and Anti-Inflammatory Activity

Oxidative stress and inflammation play crucial roles in the development of various diseases. The rich content of flavonoids, phenolic acids, and anthocyanins in *Echium* spp. underlies their strong antioxidant potential, which manifests in neutralizing reactive oxygen species and reducing lipid peroxidation [33,154]. Significant antioxidant activity is exhibited by shikonin and its derivatives, which also demonstrate anti-inflammatory potential; however, their pharmacological application may be limited due to dose-dependent cytotoxicity and safety considerations [138,145]. Studies on species in the genus have shown that extracts of *E. vulgare* show significant inhibition of pro-inflammatory cytokines such as IL-1β, TNF-α, and COX-2 [154].

The aerial parts of *E. vulgare* extracted with methanol demonstrated strong hydroxyl radical scavenging and iron-chelating abilities [101]. Good antioxidant activity was also observed in ethanolic extracts of *E. vulgare*, rich in phenols and flavonoids [33]. With its antioxidant capacity, *E. vulgare* has potential applications in improving the nutritional and sensory qualities of functional beverages, providing valuable insight for developing healthy products in beverage manufacturing [157,158]. Polysaccharides extracted from *E. vulgare* flowers could be considered to be potential new antioxidant and anti-listeriosis agents for use in biological and functional foods [159].

*Echium italicum* can also serve as a natural antioxidant source, with antioxidant activity levels varying depending on the plant’s geographical origin [160]. Anti-inflammatory activity has been observed with topical applications—extracts of *E. italicum* and *E. vulgare* have traditionally been used to treat skin inflammations, abrasions, burns, and insect bites [46,161]. Chemical analyses of *E. vulgare* honey suggest that phenolics in the yellow-golden honey are the main active components responsible for strong antioxidant and radical scavenging activities [155].

Bee-pollen extracts from *E. plantagineum* may exert anti-inflammatory effects by reducing nitric oxide and prostaglandins. The extract can scavenge reactive species, nitric oxide, and O_2_, and decreasing oxidative stress markers in cells at low concentrations [150,151,152,162,163].

### 6.2. Anxiolytic and Neuroprotective Effects

Anxiolytic and neuroprotective effects have been reported for *E. vulgare* and *E. italicum*, primarily with ethanolic extracts [37,98,164]. Low doses of the aqueous extract of the aerial parts of *E. vulgare* and high doses of the alcoholic extract have a clear antidepressant activity, but more studies in this regard should be pursued to obtain more knowledge about anti-oxylitic activity of *E. vulgare* [164]. In vitro activities indicate that extracts from flowers and leaves of *E. vulgare* may exert protective effects in vivo against oxidative damage and free radical-induced injury occurring in various pathological conditions [102]. Ethanol and aqueous extracts from *E. italicum* also show anxiolytic and sedative activities without impairing motor coordination, suggesting potential therapeutic applications for anxiety and sleep disorders [146]. Al-Snafi [147] discussed diverse bioactive compounds and the pharmacological effects of *E. italicum*, which contribute to its therapeutic potential in treating various conditions, including anxiety and inflammation. In the study by Al-Snafi [147], both ethanolic and aqueous extracts of *E. italicum* aerial parts increased open-arm exploration in the elevated plus-maze and prolonged pentobarbital-induced sleeping time in mice, without impairing motor coordination.

Given the key role of flavonoids in the anxiolytic and neuroprotective effects observed in other *Echium* species, it is plausible that *E. russicum* and *E. plantagineum* may exhibit similar pharmacological activities, potentially helping to mitigate oxidative stress and neuronal apoptosis. However, despite these promising biochemical similarities, no direct pharmacological or clinical studies have yet evaluated the anxiolytic or neuroprotective effects of these species. Therefore, these hypothesized effects remain speculative and require validation through in vitro, in vivo, and clinical studies before any firm conclusions can be drawn.

### 6.3. Antimicrobial Activity

The development of resistant bacterial strains is a major public health concern. Plant extracts, including those from *E. italicum*, represent a promising alternative for the prevention and treatment of bacterial infections [165]. Several studies confirm the bactericidal and fungicidal activity of *Echium* spp., with pronounced effects against *Staphylococcus aureus*, *Escherichia coli*, *Candida albicans*, and some strains of *Pseudomonas aeruginosa* [6].

Three different extracts (aqueous, ethanol, and methanol) obtained from *E. vulgare* were evaluated for antibacterial activity against 10 bacterial species, including *Streptococcus pyogenes*, *Staphylococcus aureus*, *Staphylococcus epidermidis*, *Escherichia coli*, *Pseudomonas aeruginosa*, *Salmonella typhimurium*, *Serratia marcescens*, *Proteus vulgaris*, *Enterobacter cloacae*, and *Klebsiella pneumoniae* using the disk diffusion method. The species exhibited inhibitory activity against both Gram-positive and Gram-negative bacteria [99,140].

Seed extracts of *E. vulgare* showed promising results against *Escherichia coli* and may serve as a potential source for antimicrobial therapies [55,100,141]. The antimicrobial activity of *E. italicum* essential oil was investigated using the disk diffusion method and minimum inhibitory concentration determination, revealing concentration-dependent antimicrobial activity against *Bacillus subtilis*, *Staphylococcus aureus*, *Escherichia coli*, *Salmonella typhi*, *Pseudomonas aeruginosa*, *Aspergillus niger*, and *Candida albicans* [69,143].

Acetone extracts of *E. italicum* demonstrated strong antimicrobial potential against *Salmonella enteritidis* and *Proteus vulgaris* [55]. Weak inhibitory activity was reported against *Helicobacter pylori*, *Mycobacterium smegmatis*, and *Mycobacterium avium* [144].

The antimicrobial effect of *E. italicum* was also assessed against *Candida albicans*, *Klebsiella pneumoniae*, *Escherichia coli*, *Bacillus megaterium*, and *Staphylococcus aureus*, with the hexane extract showing better activity than the methanol extract (IC_50_ = 20.7 µg/mL) [142].

Extracts from leaves and flowers of *E. plantagineum* exhibited activity against nematodes and trichomonads, including the flagellated protozoan *Trichomonas gallinae*, nematode *Meloidogyne javanica*, and fungus *Aspergillus niger* [153].

### 6.4. Cytotoxicity and Antitumor Activity

Although limited, data exist on the cytostatic effects of *Echium* spp. extracts on cancer cell lines such as HepG2, MCF-7, and HeLa. Methanolic and hexane extracts from *E. vulgare* seeds showed promising results against human breast cancer (MCF-7) and human liver cancer (HepG2), suggesting potential as sources for anticancer therapies [140,141]. Flavonoids and alkaloids present in the extracts are believed to induce apoptosis, cell cycle arrest, and oxidative stress in tumor cells [6].

Shikonin, used as a red pigment in food and cosmetics, exhibits cytotoxic activity against cancer cells, but its clinical use as an anticancer drug is limited due to observed toxicity. Acetylshikonin, a derivative of shikonin, shows promising antitumor potential [145].

Several studies confirm the cytotoxic activity of *E. italicum* against human rhabdomyosarcoma cells (RD) and human cervical carcinoma cells (Hep2c) [37]. It is also suggested that this species may have applications against liver cancer cell lines [142].

### 6.5. Application in Dermatology and Cosmetics

Shikonins are commercially important secondary metabolites with diverse biological activities, including antimicrobial, insecticidal, antitumor, and antioxidant properties [166]. Owing to their intense pigmentation, they are widely applied in the food, textile, and cosmetic industries. Shikonin and its derivatives, the most significant naphthoquinone pigments, are broadly distributed in Boraginaceae, including the genus *Echium* [148,149].

Seed oils of *E. plantagineum* and *E. vulgare*, rich in γ-linolenic and stearidonic acids, are used in dermocosmetic formulations for atopic dermatitis, dry and irritated skin, superficial wounds, fissures, and age-related skin changes. Although controlled clinical data remain limited, formulation and preclinical studies indicate that topical *Echium* seed oil can support epidermal barrier function and attenuate inflammatory responses [80,83,127]; its stabilization and delivery have also been optimized [123]. In addition, roots of *E. italicum* contain high levels of the purine derivative allantoin, which soothes rough and sensitive skin and may benefit sunburn [58]. Allantoin is frequently incorporated in products targeting sensitive skin, acne, rosacea, and eczema.

Antimicrobial activity represents another important dermatological property of *Echium* spp. According to Eberle et al. [79], *E. plantagineum* can also be considered to be an alternative oilseed crop in summer-wet temperate regions, with its seed oil being particularly valued in the cosmetic industry due to its high content of omega-3 fatty acids, especially stearidonic acid.

Honey derived from *Echium* spp. is traditionally used in wound-healing and skin care, benefiting conditions such as pityriasis, seborrhea, dandruff, diaper rash, psoriasis, and hemorrhoids [156]. However, it should be noted that PAs are present in the anthers of *E. vulgare* and *E. plantagineum*. These compounds are hepatotoxic, and potentially carcinogenic and genotoxic, thus highlighting the need for strict monitoring of pollen and honey intended for human consumption until appropriate processing conditions or selection strategies are established to reduce PAs content [110,167,168].

## 7. Toxicological Profile and Safety of *Echium* L. Representatives

Although species of *Echium* L. possess considerable pharmacological potential, their safety of use remains a subject of ongoing scientific debate, primarily due to the presence of pyrrolizidine alkaloids (PAs) [169]. Field et al. [170] note that not all compounds within this group exhibit the same carcinogenic potential, underscoring the need for new analytical methods and further research to enable a more accurate assessment of the concentrations and risks associated with these alkaloids.

PAs rank among the most hepatotoxic natural compounds and are widely distributed worldwide [171]. Characterized by a typical pyrrolizidine core, they are synthesized mainly as defensive metabolites against herbivores, displaying considerable structural diversity and occurrence across numerous plant species [172]. Most PAs require metabolic activation to manifest their toxicity, and exposure to herbal preparations or dietary supplements containing, or contaminated with, PAs has been associated with hepatic sinusoidal obstruction syndrome (HSOS)—a rare but often fatal condition [171].

Beyond hepatotoxicity, PAs also exhibit strong genotoxic, cytotoxic, tumorigenic, and neurotoxic activities and are recognized contaminants of both food and pharmaceutical products [173,174,175,176,177]. Although their bitterness generally deters consumption by herbivores, they remain a significant concern in feed and food products, necessitating strict regulatory oversight, particularly for products containing *Echium* spp. [178].

In various *Echium* species, including *E. italicum*, *E. plantagineum*, and *E. vulgare*, numerous PAs have been identified, such as echimidine, echiumine, echimidine-N-oxide, and intermedine [179,180]. PA analysis is challenging due to its structural diversity and low concentrations, necessitating highly sensitive analytical techniques. The choice of method critically affects detection accuracy, as conventional HPLC analysis may obscure the true alkaloid profile, leading to an underestimation of toxicity [181].

Within the body, PAs are metabolized in the liver into reactive intermediates that form covalent bonds with DNA and proteins, resulting in liver damage, including veno-occlusive disease, hepatocellular carcinoma, and sinusoidal obstruction syndrome, and are associated with mutagenic and hepatocarcinogenic effects [182,183]. Cao et al. [184] unequivocally detected echimidine in honey and mead, highlighting the need for broader studies and routine monitoring to assess long-term low-level or intermittent exposure and its implications for chronic disease risk.

Egebjerg et al. [185] and Casado et al. [186] classify *E. vulgare* and other species in the genus as potentially toxic to humans, with chronic PA accumulation considered a serious public health concern. According to Dusemund et al. [187], PA toxicity is cumulative and structure-dependent: cyclic diesters with a 7S-configuration are the most toxic, whereas monoesters with a 7R-configuration exhibit considerably lower toxicity. EFSA has established a BMDL_10_ of 237 μg/kg body weight/day as a reference for risk assessment [187].

PAs are mutagenic both in vivo and in vitro, a property closely linked to their carcinogenic potential [187]. Despite their toxicity, PAs also display a broad range of pharmacological activities that may be leveraged in drug discovery programs [188,189]. Various strategies have been proposed to reduce PA content, including selective extraction through chromatographic methods, genetic selection of low-alkaloid cultivars, and agronomic control over cultivation and harvesting practices of herbal raw materials [190].

## 8. Relevance of the Topic and Current Research Trends

**Figure 8:**
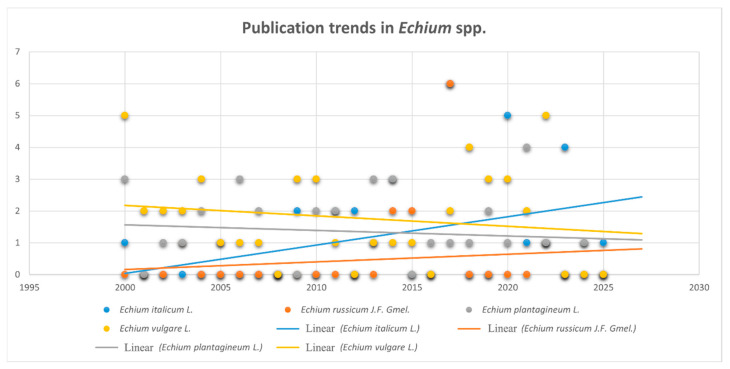
Publication trends in research connected with *E. italicum* L., *E. russicum* J.F. Gmel., *E. vulgare* L., and *E. plantagineum* L.

The data presented in this review were carefully selected and organized to provide a comprehensive overview of the phytochemical constituents and biological activities of four *Echium* species, thereby enabling a clearer understanding of their potential. This survey encompasses a total of 124 scientific studies related to the composition and properties of *E. italicum*, *E. russicum*, *E. plantagineum*, and *E. vulgare*. Figure 8 illustrates the annual distribution of publications concerning these four species over the 2000–2025 period.

The publication activity can be divided into three distinct phases, as shown in the accompanying figure:

- Phase 1 (2000–2007)—characterized by low intensity, dominated by studies on *E. vulgare* and *E. plantagineum*.
- Phase 2 (2008–2016)—a period of diversification, marked by the emergence of research on *E. italicum* and sporadic publications on *E. russicum*.
- Phase 3 (2017–2023)—a peak activity phase, with simultaneous maxima for several species, particularly in 2017.

Among the dataset, *E. vulgare* accounts for the largest cumulative number of records (n ≈ 47), representing approximately 38% of all entries. It maintains a relatively constant presence, with notable peaks in 2000, 2018, and 2022. *E. plantagineum* ranks second (n ≈ 35; 28%), receiving periodic research attention. *E. italicum* (n ≈ 30; 24%) demonstrates sustained interest, with clusters of publications between 2000–2007 and 2013–2022, including a pronounced peak in 2017 (n = 6). In contrast, *E. russicum* remains the least studied (n ≈ 12; 10%), appearing sporadically with a notable co-peak in 2017 alongside *E. italicum*.

The dominance of *E. vulgare* suggests its prioritization in research, potentially linked to its broader geographic distribution and well-documented bioactive compound profiles.

## 9. Future Perspectives and Conclusions

Species of the genus *Echium* are valuable botanical resources with significant ethnopharmacological and phytochemical potential. This review summarizes studies on *E. italicum*, *E. russicum*, *E. plantagineum*, and *E. vulgare* conducted over the past 25 years (2000–2025). Evidence supports traditional medicinal uses and highlights diverse bioactive constituents with therapeutic effects against inflammation, oxidative stress, skin disorders, anxiety, and cancer. However, the presence of toxic pyrrolizidine PAs necessitates rigorous safety evaluations.

Research remains limited, particularly in Eastern European populations, including Bulgaria, where data on bioactive compound composition, pharmacological activity, and toxicity are scarce. There is an urgent need for systematic phytochemical standardization, detailed pharmacological profiling, and comprehensive toxicological assessment. Increasing interest in *Echium* seed oils—rich in stearidonic and γ-linolenic acids—alongside the genus’s ecological role in supporting pollinators, underscores its dual potential for medicinal applications and sustainable agroecological systems.

This review also shows that the extraction and analytical assessment of PAs in plant extracts are only partially addressed, but could benefit from the development of innovative and efficient extraction schemes and advanced molecularly selective techniques for quantitative analysis, ensuring the safety of pharmaceutical products, herbal materials, and herbal extracts.

Future research should focus on risk mitigation strategies, including the development of PA-free extracts, advanced purification techniques, and stringent regulatory oversight. Clear labeling, toxicokinetic studies, and comprehensive risk assessments are recommended for products intended for internal use. Fully realizing the therapeutic potential of *Echium* species requires a multidisciplinary approach integrating phytochemistry, pharmacology, toxicology, and agricultural sciences within a robust regulatory framework.

## Figures and Tables

**Table 1:** Comparative morphological characteristics of *Echium* species naturally distributed in Bulgaria.

Characteristic	*E. italicum*	*E. russicum*	*E. plantagineum*	*E. vulgare*
life form	biennial	biennial	annual–biennial	biennial–short-lived perennial
stem height (cm)	40–150	25–80	20–60	20–100
stem indumentum	appressed simple hairs + bristly hairs on tubercles	stiff hairs on small tubercles	bristly hairs with bulbous bases	stout spreading bristles + fine appressed hairs
basal leaves	lanceolate, 20–35 × 1.5–4.0 cm	linear-lanceolate, 2–11 × 0.7–1.2 cm	ovate, 3–12 × 1.2–1.5 cm	oblanceolate, 2–25 × 0.5–3 cm
cauline leaves	narrowly elliptic	narrow, linear-lanceolate	narrow-lanceolate, cordate base	linear-lanceolate, sessile
inflorescence	branched, pyramidal	narrow, spike-like	broad, paniculate or racemose	panicle of helicoid cymes
corolla size and color	10–12 mm, whitish to blue-violet	8–12 mm, dark red	15–20 mm, blue to deep blue (rarely white)	10–20 mm, pink in bud, bright blue at anthesis
stamens	4–5 exserted	exserted, longer than corolla	2 shortly exserted, 3 included	exserted
nutlet size (mm)	2.8–3.8 × 2.3–2.5	1.8–3.2 × 1.5–1.8	2.0–3.0 × 2.0–2.3	1.8–3.2 × 1.5–1.8

**Table 2:** Traditional medicinal applications of *Echium* species.

Species	Traditional Uses	Plant Parts Used	Mode of Application	Geographic Region	References
***Echium italicum***	anti-inflammatory, antiseptic, analgesic, depurative, diuretic, emollient for respiratory infections, sudorific, treatment of burns, wounds, abscesses	leaves, roots	decoctions, infusions, ointments with oil, poultices	Anatolia, Southern Europe (Italy, Croatia, Montenegro), Turkey	[35,36,37,38,39,40,41,42]
***Echium russicum***	anemia, fatigue, gynecology, hemorrhoids, osteomyelitis, snake bites, wound-healing	leaves, flowers	tea, macerations, poultices	Eastern Europe, Georgia, Turkey	[36,43,44]
***Echium plantagineum***	chest pain, cough, fever, inflammation, insect bites, muscle strain, skin conditions, urinary tract infections	leaves, flowers, seeds	pastes, tea, syrup, topical oil	Africa, America, Asia, Australia (introduced use), Eastern Europe, Europe, and Oceania, Iberian Peninsula, Mediterranean, North Africa	[13,33,45]
***Echium vulgare***	anti-inflammatory, balsamic agents, blood purifier, cough suppressant, depuratives, diuretic, epilepsy, expectorant, fever, gynecological disorders, lactogenic remedies, laxative effects, muscle strain, snake bites, ulcers, urinary tract infections, wound-healing	bark, flowering tops, flowers, leaves, root, seeds, stem	compresses, decoction, infusion (tea), ointments, poultices, syrups	Africa, America, Asia, Balkans, Bulgaria, Central and Eastern Europe, Europe and Oceania, Mediterranean, Morocco, Serbia, Turkey	[13,24,36,41,45,46,47,48,49,50,51]

**Table 3:** Determined Bioactive Compounds and Pharmacological Properties of the Studied Species of the Genus *Echium*.

Class of Compound	Reported Compounds	Analytical/Isolation Methods	Pharmacological Properties	References
***Echium italicum***				
Phenolic compounds	caffeic acid, chlorogenic acid, ferulic acid, p-coumaric acid, hydrocaffeic acid, p-hydroxybenzoic acid, rosmarinic acid, sinapic acid, tannins	Folin–Ciocalteu colorimetric assay; UV–Vis spectrophotometry (λ = 765 nm); HPLC-DAD (C18 column, multiwavelength detection); PVP-Folin–Ciocalteu method	antibacterial, antimicrobial, antioxidant, radiation protection	[37,41,54,55,56,57]
Flavonoids	anthocyanins, apigenin, apigenin glycoside, kaempferol, luteolin glycoside, myricitrin, naringenin, quercetin, rutin	Aluminum chloride (AlCl_3_) spectrophotometric method (λ = 415 nm); HPLC-DAD; HPCE-UV–Vis DAD (190–600 nm)	anxiolytic, sedative	[37,41,54,55,56,57,58]
Fatty acids	arachidonic acid, caproic acid, erucic acid, heptadecanoic, lauric acid, linoleic acid, myristic acid, oleic acid, stearidonic acid, α-linolenic acid, γ-linolenic acid, palmitic acid, palmitoleic acid, pentadecanoic, stearic acid	Gas Chromatography (GC) with Flame Ionization Detector (FID); GC-MS; FAME (Fatty Acid Methyl Ester) derivatization	anti-inflammatory	[54,59,60,61,62]
Pyrrolizidine alkaloids	echimin, echinin, leptanthine, lycopsamine, uplandicine	LC–MS/MS (Liquid Chromatography-Tandem Mass Spectrometry)	potential toxicity	[63]
Quinones	2-methyl-n-butyryl shikonin, 3,3-dimethyl acrylyshikonin, acetyl shikonin, alkanin, angeloylshikonin, deoxyshikonin, isobutyryl shikonin, isovalerylshikonin, propionyl shikonin, shikonin	HPCE (High-Performance Capillary Electrophoresis) with UV–Vis DAD; HPLC-VIS/MS (APCI-mode); TLC and preparative HPLC; ^1^H/^13^C NMR	antibacterial, anti-allergic, antimicrobial, antioxidant, antitumor, antithrombotic, wound-healing	[58,64,65,66,67,68]
Terpenes	limonene, pulegone	GC, GC-MS	antimicrobial, aromatherapeutic potential	[69]
Phytosterols	24-methylenecholesterol, β-sitosterol, δ5,23-stigmastadienone, δ5-avenasterol, campesterol, clerosterol, stigmast-4-ene-3,6-dione, sitostanol, stigmasterol	GC-FID (after silylation); HPLC; Preparative TLC + ^1^H/^13^C NMR	cardiovascular protection	[6,61,70]
***Echium russicum***				
Phenolic compounds	caffeoylquinic acid, chlorogenic acid, globoidnan a, rabdossin (disodium salt), rosmarinic acid, tannins	Folin–Ciocalteu colorimetric assay; PVP-Folin–Ciocalteu method; Capillary Zone Electrophoresis (CZE), UV detection	antioxidant, hepatoprotective, radiation protection	[54,58,71,72]
Flavonoids	anthocyanins, apigenin-7-o-glucoside, naringin, rutin, anthocyanins	HPCE (High-Performance Capillary Electrophoresis) with UV–Vis DAD (190–600 nm)	anti-inflammatory, antiviral	[54,58]
Fatty acids	arachidonic acid, linoleic acid, oleic acid, stearidonic acid, α-linolenic acid, γ-linolenic acid, palmitic acid, stearic acid	Gas Chromatography-Mass Spectrometry (GC-MS); Gas-Liquid Chromatography (GLC)	anti-inflammatory	[54,73]
Pyrrolizidine alkaloids	No specific data were found for the species.	-	-	-
Quinones	shikonin	Capillary Zone Electrophoresis (CZE), UV detection	anti-inflammatory, antimicrobial, antitumor, wound-healing	[44,58,71,72]
Terpenes	No specific data were found for the species.	-	-	-
Phytosterols	No specific data were found for the species.	-	-	-
***Echium plantagineum***				
Phenolic compounds	caffeic acid, globoidnan a, ferulic acid, rosmarinic acid, sinapic acid, γ-tocopherol, rabdosiin	1D/2D NMR spectroscopy; HRMS; GC-FID; HPLC-DAD; UV–Vis spectrophotometry (Folin–Ciocalteu assay)	antioxidant, anticancer	[74,75]
Flavonoids	petunidin-3-o-rutinoside; delphinidin; cyanidin; peonidin; malvidin-3-o-rutinoside and cyanidin-3-(6″-malonylglucoside), kaempferol, quercetin, rutin	HPLC-DAD; HPLC-MS (ESI mode); LC–MS/MS	anti-allergic, photoprotective	[67]
Fatty acids	α-linolenic acid, γ-linolenic acid, caproic acid, caprylic acid, capric acid, stearidonic acid, undecanoic acid	GC-FID; GC-MS; GLC-FID; GC-MS (FAME derivatization); NMR; Silver-ion HPLC; TLC-FID	anti-inflammatory	[15,16,17,53,75,76,77,78,79,80,81,82,83,84,85]
Pyrrolizidine alkaloids	3′-o-acetylechiumine-n-oxide, 3′-o-acetylintermedine [sol] lycopsamine, 7,9-ditigloylretronecine n-oxide, 9-o-angelyl retronecine-n-oxide, acetyl lycopsamine, echimin, echimidine, echimidine n-oxide, echimiplatine-n-oxide, echinin, echi-uplatine-n-oxide, echiumine, echiumine n-oxide, intermedine, leptanthine-n-oxide, lycopsamine, lycopsamine n-oxide, retrorsine	UHPLC-QTOF-MS; LC-ESI/MS; GC-MS; SPE (solid-phase extraction, cation-exchange); LC–MS/MS (QTRAP)	potentially hepatotoxic, hepatotoxic photosensitization	[86,87,88,89]
Quinones	1,8-dihydroxy-3-methylanthraquinone, acetylshikonin, angelicshikonin, dimethylacrylshikonin, isovalerylshikonin, shikonin	UHPLC–QTOF-MS; LC-ESI/MS; GC-MS; Spectrophotometry (Nanodrop 2000c, λ = 493–562 nm); Ethanolic extraction	antibacterial, wound-healing	[15,89,90,91,92,93,94,95]
Terpenes	β-carotene	HPLC-DAD; HRMS; Spectrophotometric β-carotene quantification; Oil extraction (hydraulic, solvent, cold-press)	antioxidant	[75]
Phytosterols	β-sitosterol, campesterol	HPLC; HRMS; Spectrophotometric quantification of sterols/tocopherols; Oil extraction and oxidative stability testing	cardiovascular protection	[75]
***Echium vulgare***				
Phenolic compounds	3-(3′,4′-dihydroxyphenyl)-(2R)-lactic acid, caffeic acid, catechol, chlorogenic acid, cis-cinnamic acid, ellagic acid, ferulic acid, gallic acid, hydrocaffeic acid, isoferulic acid, p-coumaric acid, protocatechuic acid, rosmarinic acid, salicylic acid, tannins, vanillic acid	Ethanolic maceration (70–96% MeOH/EtOH); Folin–Ciocalteu spectrophotometric assay; UV–Vis (DPPH, Fe^2+^-Ferrozine, β-carotene test); Capillary Zone Electrophoresis (CZE); Column chromatography (SiO_2_, Sephadex LH-20); ^1^H/^13^C NMR, COSY, HMQC, HMBC; ESI-MS	anti-inflammatory, antioxidant, antimicrobial	[41,71,96,97,98,99,100,101,102]
Flavonoids	apigenin, hesperetin, hesperidin, kaempferol, kaempferol 3-o-neohesperidoside, naringen, naringin, quercetin, quercetrin, rutin	MeOH maceration; UV–Vis spectrophotometry (AlCl_3_ colorimetric assay at 415 nm); HPLC-DAD; HPCE/CZE (UV–VIS DAD); ^1^H/^13^C NMR, COSY, HMQC, HMBC; ESI-MS	anticonvulsant, neuroprotective	[41,58,98,99,100,101,102,103]
Fatty acids	α-linolenic acid, γ-linolenic acid, stearidonic acid	GC-FID; GC-MS; GLC-MS; HPLC; Silver-ion TLC (Ag^+^-TLC); Supercritical CO_2_ (SC-CO_2_) oil extraction; NMR	anti-inflammatory	[16,17,73,104,105,106,107,108]
Pyrrolizidine alkaloids	7-o-acetylvulgarin, echiimin, echimidine, echinin, echinatine, echivulgarine, intermedin, leptanthine, lycopsamine, uplandicine, vulgarin	MeOH extraction; Aqueous acid extraction; Strong cation-exchange solid-phase extraction (SCX-SPE); LC–MS/MS; LC-HR-MS (Orbitrap, QTOF); UHPLC-MS/MS (TSQ Quantiva); ^1^H/^13^C NMR	potentially toxic	[63,87,100,109,110,111,112,113]
Quinones	acetylshikonin, dimethylacrylshikonin, isovalerylshikonin, shikonin	Ethanolic extraction; Capillary Zone Electrophoresis (CZE, UV–VIS DAD); UHPLC-QTOF-MS; Spectrophotometry (Nanodrop 2000c, λ = 493–562 nm)	anti-inflammatory, antimicrobial, antitumor, wound-healing	[32,58,71,90,98]
Terpenes	α-bisabolol, camphor, caryophyllene oxide isomers, cis-geranyl acetate, endo-borneol, lavandulyl acetate, linalool, trans-geraniol, trans-geraniol acetate, α-terpineol	GC; GC-MS; HPCE with chemometric analysis; CZE	anti-inflammatory, antimicrobial, antitumor, aromatherapeutic potential, emollient, soothing	[114]
Phytosterols	β-sitosterol, campesterol, sterone stigmast-4-ene-3,6-dione, stigmasterol, sitostanol	Column chromatography (silica gel; CHCl_3_, Et_2_O, EtOAc, Me_2_CO); Preparative TLC; GC-MS; HPLC; ^1^H/^13^C NMR, INEPT	cardiovascular protection	[6,70,108,115]

Note: Analytical methods listed correspond to those reported in the cited references; abbreviations follow IUPAC guidelines.

**Table 4:** Summary of the main pharmacological activities of *Echium* spp.

Species	Type of Extract/Active	Experimental Model	Observed Effects	References
*E. italicum*	Acetone extract	In vitro antibacterial	Strong activity vs. *S. enteritidis*, *P. vulgaris*	[55]
*E. italicum*	Methanolic/hexane seed extracts; crude extracts	Cancer cell lines (MCF-7, HepG2, RD, Hep2c)	Cytotoxic/antitumor effects	[37,140,141,142]
*E. italicum*	Essential oil	Disk diffusion; MIC assays	Concentration-dependent activity vs. *B. subtilis*, *S. aureus*, *E. coli*, *S. typhi*, *P. aeruginosa*, *A. niger*, *C. albicans*	[69,143]
*E. italicum*	Various extracts	In vitro antibacterial	Weak inhibition vs. *H. pylori*, *M. smegmatis*, *M. avium*	[144]
*E. italicum*	Shikonin/derivatives (incl. acetylshikonin)	In vitro (multiple cancer models); pharmacology reviews	Anticancer/antibacterial/wound-healing; acetylshikonin with antitumor potential	[57,65,138,145]
*E. italicum*	Aqueous and ethanolic (aerial parts)	Mice: elevated plus-maze; pentobarbital sleep	Anxiolytic and sedative without motor impairment	[146,147]
*E. italicum* (roots)	Allantoin; shikonin pigments	Dermatology/cosmetics context; phytochemical reports	Soothing/regenerative (allantoin); antioxidant/antimicrobial pigments	[58,148,149]
*E. plantagineum*	Bee-pollen extracts	RAW 264.7 macrophages; basophils; Caco-2 cells	Reduction of NO, iNOS, and COX-2 mediators; effects on degranulation; antioxidant protection	[150,151,152]
*E. plantagineum*	Leaves/flowers extracts	In vitro antiparasitic/antifungal (nematodes, *Trichomonas gallinae*, *A. niger*)	Antinematodal/antitrichomonad/antifungal activity	[153]
*E. plantagineum*, *E. vulgare* (seed oil)	Seed oils rich in GLA/SDA	Cosmetic/dermatological applications (reported); formulation/processing studies	Anti-inflammatory/skin-barrier support (reported); high ω-3 content relevant for cosmetics/nutraceuticals	[80,83,127]
*E. vulgare*	Methanolic extract (aerial parts)	In vitro antioxidant assays	Strong OH scavenging; iron-chelating capacity	[101]
*E. vulgare*	Ethanolic extracts (various parts)	In vitro antioxidant assays; phenolics/flavonoids profiling	Good antioxidant activity consistent with high phenolic/flavonoid content	[33]
*E. vulgare*	Crude extracts (unspecified)	Animal model (hyperlipidemia)	Improved blood parameters, lipid profile, liver function; histopathology improvement	[98]
*E. vulgare*	Extracts (unspecified)	In vitro/ex vivo inflammatory readouts	Inhibition of IL-1β, TNF-α, COX-2	[154]
*E. vulgare*	Aqueous/ethanol/methanol extracts	Disk diffusion vs. 10 bacteria (Gram±)	Broad antibacterial inhibition	[99,140]
*E. vulgare* (seeds)	Seed extracts	Antimicrobial screens (incl. *E. coli*)	Antimicrobial activity; notable activity against *E. coli*	[55,100,141]
*Echium* spp. honey	Phenolic-rich honey	In vitro antioxidant assays; dermal uses (review)	Antioxidant, radical scavenging; traditional wound/skin care uses	[155,156]

MIC—minimum inhibitory concentration.

## Data Availability

No new data were created or analyzed in this study. Data sharing is not applicable to this article.
